# Oral Supplementation with Cholecalciferol 800 IU Ameliorates Albuminuria in Chinese Type 2 Diabetic Patients with Nephropathy

**DOI:** 10.1371/journal.pone.0050510

**Published:** 2012-11-29

**Authors:** Yan Huang, Haoyong Yu, Junxi Lu, Kaifeng Guo, Lei Zhang, Yuqian Bao, Haibing Chen, Weiping Jia

**Affiliations:** Shanghai Diabetes Institute, Shanghai Key Laboratory of Diabetes Mellitus, Shanghai Clinical Center for Diabetes, Department of Endocrinology and Metabolism, Shanghai Jiaotong University Affiliated Sixth People’s Hospital, Shanghai, China; University of Hong Kong, China

## Abstract

**Background:**

Low vitamin D levels can be associated with albuminuria, and vitamin D analogs are effective anti-proteinuric agents. The aim of this study was to investigate differences in vitamin D levels between those with micro- and those with macroalbuminuria, and to determine whether low dose cholecalciferol increases vitamin D levels and ameliorates albuminuria.

**Methods:**

Two studies were performed in which 25-OH vitamin D_3_ (25(OH)D_3_) concentrations were determined by electrochemiluminescence immunoassay: 1) a cross-sectional study of patients with type 2 diabetes mellitus (T2DM) (n = 481) and healthy controls (n = 78); and 2) a longitudinal study of T2DM patients with albuminuria treated with conventional doses, 800 IU, of cholecalciferol for 6 months (n = 22), and a control group (n = 24).

**Results:**

1) Cross-sectional study: Compared to controls and T2DM patients with normoalbuminuria, serum 25(OH)D_3_ concentrations were significantly lower in patients with macro-albuminuria, but not in those with micro-albuminuria. Serum 25(OH)D_3_ levels were independently correlated with microalbuminuria. 2) Longitudinal study: Cholecalciferol significantly decreased microalbuminuria in the early stages of treatment, in conjunction with an increase in serum 25(OH)D_3_ levels.

**Conclusions:**

Low vitamin D levels are common in type 2 diabetic patients with albuminuria, particularly in patients with macroalbuminuria, but not in those with microalbuminuria. Conventional doses of cholecalciferol may have antiproteinuric effects on Chinese type 2 diabetic patients with nephropathy.

## Introduction

Diabetes has become a major public health problem in China, and a large-scale epidemiological survey revealed a prevalence of 9.7% approximately 5 years ago [1]. Diabetic nephropathy (DN) is the most common microvascular complication and is a major cause of end-stage renal disease that requires dialysis and/or renal transplantation [2]. Thus, strategies aimed at the treatment of DN are as important as those that target diabetes itself. Despite treatment with renin-angiotensin-aldosterone system (RAAS) inhibitors and compliance with conventional therapy for glycemic and blood pressure control, for some patients satisfactory control of urinary albuminuria is not achieved. Residual proteinuria is tightly associated with progression of renal disease, and thus additional treatments are needed for this group of patients [3].

Epidemiological studies have shown that low 25(OH)D_3_ levels are common in patients with albuminuria (spot urinary albumin/creatinine ratio (ACR) ≥30 mg/g) [4], [5]. The selective vitamin D receptor (VDR) activator, paricalcitol, effectively reduces proteinuria in patients with type 2 diabetes mellitus (T2DM) who have been treated with RAAS inhibitors [6]. Furthermore, recent research suggests that a large dose of cholecalciferol, 40,000 units weekly, is as effective as vitamin D analogs [7].

Albuminuria can be divided into two phases, micro- and macro-albuminuria. Patients with macroalbuminuria which is a more serious stage of DN, has a poor prognosis. Whether serum 25(OH)D_3_ levels can indicate the severity of DN in patients with micro and macro-albuminuria hasn’t been reported yet. Since high dose and long-term of vitamin D administration may cause a variety of side effects such as electrolyte imbalance problem, whether regular doses of vitamin D_3_ can improve proteinuria level either. There is no report about them. Thus this study is to observe the differences in serum 25(OH)D_3_ levels between patients with microalbuminuria and macroalbuminuria, and to investigate the effects of nutritional vitamin D supplementation with low dose of cholecalciferol, in addition to RAAS inhibitors, in Chinese patients with DN.

## Subjects and Methods

### Study Design

All subjects gave written informed consent, and the study was approved by the ethics committee of Shanghai Jiao Tong University Affiliated Sixth People’s Hospital and complied with the Declaration of Helsinki.

The protocol included a cross-sectional study and an open-label longitudinal study.

All patients in both studies were recruited from the outpatient clinic at the Shanghai Clinical Center for Diabetes (Shanghai, China) from January, 2011 to April, 2012. At the same time, seventy-eight healthy patients were enrolled as normo-glycemic control subjects without any history of kidney diseases or current urinary tract infection and have no supplements with vitamin D, active vitamin D analogs, or any steroid. Patients with diabetes were categorized as those with normo-albuminuria (NA), when the ACR was persistently <30 mg/g (n = 261), those with microalbuminuria (MA), when the ACR was between 30 and 300 mg/g (n = 154), and those with DN, if they had persistent albuminuria (>300 mg/g) (n = 66), without any other kidney or renal tract disease. Among them, patients were eligible if they were older than 20 years, with T2DM and ACR persistently >30 mg/g, and on stable doses of ACE inhibitors or angiotensin receptor blockers (ARBs) for 3 months or more, without supplement with vitamin D, active vitamin D analogs, or any steroid, serum parathyroid hormone concentration of 25–500 ng/L and serum calcium concentration of less than 2.45 mmol/L were enrolled into the longitudinal intervention study [6]. 46 patients entered in the longitudinal study, 22 patients received cholecalciferol (Xiamen Shark Pharmaceutical Company, orally) daily at a dose of 800 IU/d over a 6-month period, another 24 patients were classified as control group according to gender and age. Microalbuminuria were monitored at 2, 3, 4.5 and 6 months and serum 25(OH)D_3_ were measured before and after treatment. Patients continued to receive their usual diabetes care.

Demographic and clinical data, including age, sex, duration of diabetes, weight, height, and medication, were recorded. Blood pressure (BP) was measured twice with a Hawksley sphygmomanometer after 10 minutes of supine rest. The ACR was determined in three consecutive spot urine samples using the Dade Behring Nephelometer II System (antiserum to human albumin, Siemens Healthcare Diagnostics). The estimated glomerular filtration rate (eGFR) was calculated using the Modification of Diet in Renal Disease study equation (MDRD) [8].

### Electrochemiluminescence Immunoassay

Serum samples were maintained at −70°C for subsequent assays. Serum 25(OH)D_3_ measurements were performed using a commercially available electrochemiluminescence immunoassay (ECLIA) (Roche Diagnostics GmbH), according to the manufacturer’s protocol. The detection limit of human serum 25(OH)D_3_ assay was 4 ng/ml. Duplicate measurements were obtained for all samples.

### Statistical Analysis

Each variable was assessed for normal distribution. Data are expressed as mean ± standard deviation (SD) for normally distributed variables, and as median with the interquartile range for skewed variables. Skewed variables were natural logarithm-transformed to improve normality prior to analysis, and then re-transformed to their natural units in order to present them in a tabulated form. Characteristics of subjects across different patient groups were compared by ANOVA and analysis of covariance, and those between control and patient groups were compared using the t-test. Comparisons between groups before and after cholecalciferol treatment were undertaken with the Wilcoxon signed-ranked test. Pearson correlation tests, multivariable linear regression analyses, and partial correlation analyses were also performed. Only variables that were significantly (P<0.05) related to 25(OH)D_3_ by Pearson correlation analyses were entered into the multiple linear stepwise regression analysis. All calculations were undertaken using GraphPad Prism software (GraphPad; San Diego, CA) and the statistical package for social sciences (SPSS) 17.0 software (Los Angeles, CA). All reported P-values were 2-tailed, and P-values <0.05 were considered statistically significant.

## Results

General characteristics and clinical parameters of the cross-sectional study are summarized in Table 1. Compared with controls, patients with T2DM had higher BP levels, and higher levels of hemoglobin A1c (HbA1c), fasting plasma glucose (FPG), and 2-hour postprandial plasma glucose (P2hPG). Compared with the NA group, the MA and DN groups had higher systolic BP levels and body mass indices (BMI), and were older. There was no significant difference in blood glucose levels and lipid counts in patients with diabetes. There were slightly more men than women in the diabetes groups. MDRD in the DN group was lower than that observed in the control or non-DN groups.

**Figure 1:**
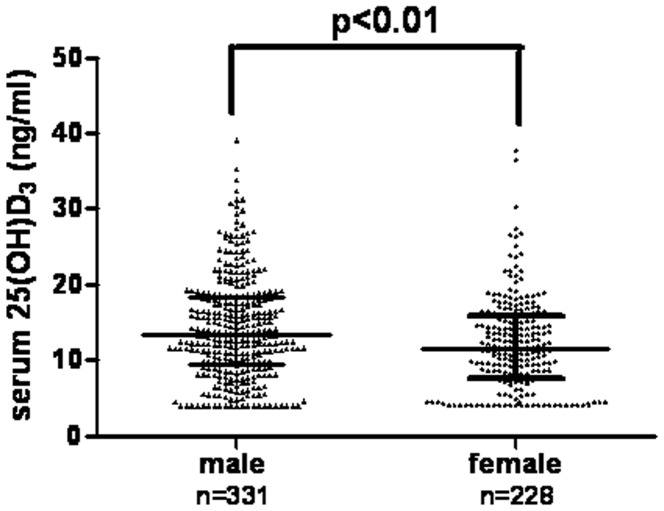
The concentrations of 25(OH)D_3_ in males (n = 331) and females (n = 228) : males: 13.34 (9.28, 18.17) ng/ml; females: 11.62 (7.58, 15.81) ng/ml (P<0.01).

**Table 1:** General and clinical parameters of healthy control subjects and type 2 diabetic patients.

			Type 2 diabetes	
	Control subjects	Normoalbuminuria	Microalbuminuria	Macroalbuminuria
Case (M/F)	78 (29/49)	261 (171/90)	154 (87/67)	66 (44/22)
Age (years)	51.62±13.10	54.61±15.47	61.36±11.47* †	59.31±11.76* †
SBP(mmHg)	129±18	131±17	141±23* †	146±21* † ‡
DBP(mmHg)	80±11	80±10	82±11	86±10* † ‡
BMI(Kg/m2)	23.76±2.58	24.24±3.36	24.99±3.7* †	26.28±4.3* † ‡
HbA1c(%)	5.30±0.25	8. 40±2.00*	8.42±1.92*	8.42±1.94*
FPG(mmol/l)	5.01±0.34	8.65±3.43*	8.51±3.89*	8.38±2.90*
PG2H(mmol/l)	6.14±1.02	13.16±4.66*	12.88±4.47*	13.40±5.66*
Antidiabetic drugs n(%)				
Monotherapy		163 (62.5%)	80 (51.9%)	16 (24.2%)
Two-drugs		60 (23.0%)	46 (29.9%)	13 (19.7%)
≥Three-drugs		38 (14.5%)	28 (18.2%)	37 (56.1%)
TC(mmol/l)	4.97±0.84	4.88±1.02	4.90±1.07	5.55±1.46* † ‡
LDL-c(mmol/l)	3.32±0.83	2.97±0.86*	2.99±0.95*	3.16±1.18
TG(mmol/l)	1.57±1.01	1.70±1.67	1.86±1.34	2.66±2.16* † ‡
HDL-c(mmol/l)	1.33±0.28	1.26±0.41	1.18±0.29*	1.15±0.40*
Antilipidemic drug				
Statins		16 (6.1%)	24 (15.6%)	14 (21.2%)
Fibrats		2 (0.8%)	6 (3.9%)	4 (6.1%)
Cr(mmol/l)	69.06±12.47	68.47±16.96	71.17±26.23	122.47±75.86 * † ‡
ACR(mg/g)	6.74(4.82–10.71)	7.65(5.06–12.02)	71.22(42.16–127.43)	1078.58(552.91–2489.17)
MDRD	116.66±25.31	122.16±43.29	118.43±44.64	76.22±40.14* † ‡
Hypertention(%)	23(29.5%)	112(42.9%)	97(63.0%)	59(89.4%)
Anti-hypertention medication				
RAS inhibitor (+)		49 (18.8%)	85 (55.2%)	46 (69.7%)
HOMA-IR	1.42±0.57	1.73±0.81	2.30±4.58*	2.45±1.36*

* *P*<0.05; vs. control;

† *P*<0.05, vs. normoalbuminuria;

‡ *P*<0.05, vs. microalbuminuria.

Median 25(OH)D_3_ concentrations in our sample were significantly higher in men than in women (13.34 (9.28, 18.17) ng/ml vs. 11.62 (7.58, 15.81) ng/ml; P<0.01) (Figure 1).

### Serum 25(OH)D_3_ Levels were Significantly Decreased in the DN Group, but not in the MA Group

**Figure 2:**
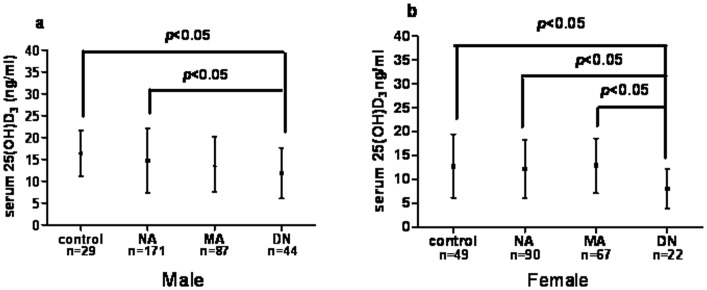
The concentrations of serum 25(OH)D3 in different groups classified according to albuminuria: normo-albuminuria (NA), micro-albuminuria (MA), and diabetic nephropathy (DN) in males (a) and females (b).

Both in male and female, mean serum 25(OH)D_3_ levels were significantly lower than in the control group (11.37 (6.48, 14.06) ng/ml vs. 15.82 (13.13, 20.29) ng/ml) and 7.57 (4.00, 10.73) ng/ml vs. 11.79 (8.07, 16.52) ng/ml, P<0.05 and 0.05, respectively) (Figures 2a and 2b). In addition, vitamin D levels were significantly lower in the DN group than in the NA group ((11.37 (6.48, 14.06) ng/ml vs. 13.79 (9.20, 18.65) ng/ml and 7.57 (4.00, 10.73) ng/ml vs. 11.93 (7.58, 16.20) ng/ml, P<0.05 and 0.05, respectively). There was no significant difference in vitamin D levels between the control and the NA groups. Of note is that serum 25(OH)D_3_ concentrations in the MA group were not significantly lower than those observed in the control or NA groups.

**Figure 3:**
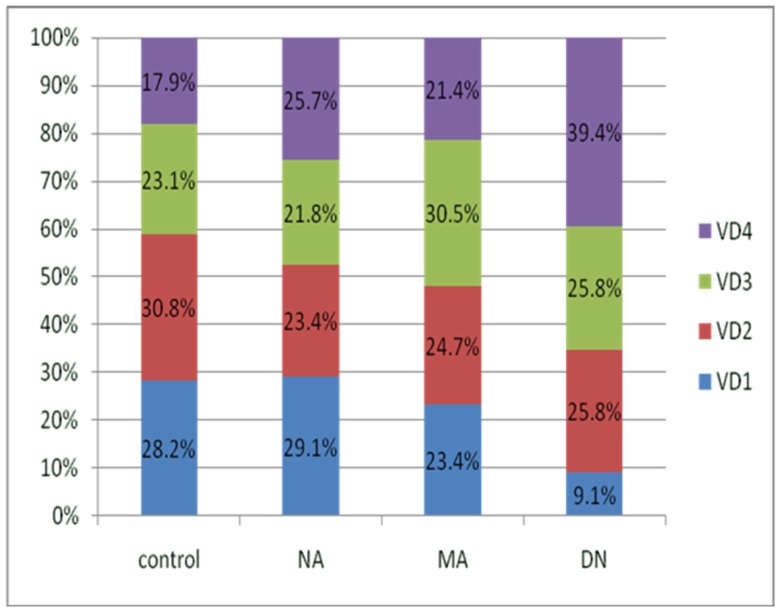
Disparity in the lower (VD4) and the upper(VD1) quartiles of 25(OH)D_3_ in the control group and in different patient groups classified according to albuminuria: normo-albuminuria (NA), micro-albuminuria (MA), and diabetic nephropathy (DN). (χ^2^ = 10.458, P = 0.015 for VD4; χ^2^ = 11.900, P = 0.008 for VD1.).

To further investigate the relationship between DN and serum 25(OH)D_3_, we divided the study population into quartiles based on serum 25(OH)D_3_ concentrations (8.56, 12.50, 17.15 ng/mL), with quartile 1 representing individuals with the lowest levels of 25(OH)D_3_ (Figure 3). In the DN group, 39.4% had 25(OH)D_3_ levels in the lower quartile, compared to 17.9% in the control group (Χ^2^ = 10.458, P = 0.015). We observed similar results with distribution of the upper quartile: 28.2% in the control group and 9.1% in the DN group (Χ^2^ = 11.900, P = 0.008).

### Vitamin D Concentration was Independently Correlated with ACR

As vitamin D levels were lower in patients with DN, we performed a correlation analysis which including all subjects to investigate related factors. The Pearson correlation analysis suggested that serum 25(OH)D_3_ levels were significantly correlated with age, BMI, fasting plasma glucose (FPG), high density lipoprotein-cholesterol (HDL-c), fasting C-peptide (OFCP), PTH and ACR in males, while in females, serum 25(OH)D_3_ levels were significantly correlated with age, glutamate transaminase (ALT), glutamic-oxal(o)acetic transaminase (AST), potassium (K ), Sodium (Na), phosphorus (Pi), PTH and ACR (Table 2).

**Table 2:** The related factors with serum 25(OH)D_3_ in male and female.

	male		female	
variables	r	P	r	P
Age	0.187	0.001**	0.204	0.002**
BMI	0.139	0.012*	−0.018	0.787
SBP	0.027	0.626	−0.054	0.422
HbA1c	−0.108	0.059	−0.036	0.599
OFG	−0.113	0.042*	−0.039	0.556
TC	−0.038	0.494	−0.031	0.640
TG	−0.001	0.979	−0.029	0.668
HDL	−0.175	0.002**	−0.010	0.879
LDL	0.032	0.571	−0.018	0.786
lnCRP	−0.063	0.263	−0.048	0.473
ALT	−0.005	0.924	0.137	0.040*
AST	−0.022	0.698	0.178	0.008**
BUN	0.053	0.344	0.006	0.928
Cr	−0.041	0.462	−0.114	0.087
UA	0.058	0.297	0.040	0.550
MDRD	−0.064	0.251	0.004	0.956
K	−0.059	0.335	−0.192	0.014*
Na	−0.046	0.475	−0.171	0.048*
Cl	0.097	0.133	−0.097	0.264
Ca	−0.072	0.242	−0.150	0.058
Pi	0.026	0.679	−0.250	0.001**
lnPTH	−0.168	0.002**	−0.200	0.002**
lnACR	−0.138	0.012*	−0.186	0.005**
lnHOMA-IR	−0.084	0.457	−0.004	0.980

* *P*<0.05.

** *P*<0.01.

In order to elucidate independent relationships between vitamin D and clinical parameters, we selected 25(OH)D_3_ as a dependent variable and other clinical parameters as the independent variables, thereby building a multiple linear stepwise regression equation. Only variables that were significantly (P<0.05) related to 25(OH)D_3_ by Pearson correlation analyses were entered into the multiple linear stepwise regression analysis. The results revealed an independent correlation between 25(OH)D_3_ and uACR (β = −0.290, P<0.01) in men, and the other four independent parameters were age (β = 0.191, P = 0.001), BMI (β = −0.168, P = 0.007), lnPTH (β = −0.133, P = 0.018) and HDL (β = −0.133, P = 0.028). In women, uACR did not affect 25(OH)D3 levels the most, and the parameters were age (β = 0.224, P = 0.004), lnPTH (β = −0.233, P = 0.002), P (β = 0.194, P = 0.013), lnACR (β = −0.184, P = 0.016).

### ACR Levels Significantly Decreased in the Early Phase of Treatment with Cholecalciferol 800 IU Daily

We demonstrated in our cross-sectional study that patients with DN had low vitamin D levels compared to controls. As previously reported, vitamin D analogs or high dose of cholecalciferol are useful as anti-proteinuric agents, and we hypothesized that a conventional low dose of cholecalciferol, 800 IU daily, would also be effective.

A total of 46 patients entered the 6-month follow-up period, 22 in the treated group and 24 in the control group. Baseline patient characteristics are shown in Table 3. Compared to the control group, the treated group had better blood glucose control. Mean MDRD levels were a little lower and mean calcium levels were higher in the treated group. Median 25(OH)D_3_ concentrations were 14.45 (8.73, 18.71) ng/ml and 14.04 (8.73, 20.96) ng/ml in the treated group and in the control group, respectively.

**Table 3:** Baseline patient characteristics.

	Treated group	Untreated group	P value
**Age**	61.1±10.4	60.0±12.2	0.67
**Cases(male)**	22(14)	24(14)	0.77
**Duration of diabetes mellitus(years)**	10±6.1	12±7.7	0.17
**SBP(mmHg)**	138±21	140±21	0.87
**DBP(mmHg)**	79±10	81±7	0.31
**FPG(mmol/l)**	6.5±1.3	8.2±3.0	0.02
**HbA_1_C(%)**	7.1±1.4	8.2±1.3	0.01
**TC(mmol/l)**	5.05±1.1	4.87±1.2	0.60
**TG(mmol/l)**	2.31±1.3	2.17±2.0	0.78
**HDL-c(mmol/l)**	1.08±0.3	1.16±0.3	0.55
**LDL-c(mmol/l)**	2.8±0.7	2.6±0.8	0.43
**Ca(mmol/l)**	2.32±0.1	2.25±0.1	0.02
**Pi(mmol/l)**	1.29±0.2	1.25±0.17	0.60
**PTH(ng/l)**	33.5±15.5	29.71±10.5	0.35
**MDRD**	87±33.01	108±32.93	0.04
**25(OH)D_3_(ng/ml)**	14.4 (8.72,18.71)	13.4(8.06,17.61)	0.79
**ACR(mg/g)**	97.4 (62.43,476.70)	114.4 (65.15,324.57)	0.92

**Figure 4:**
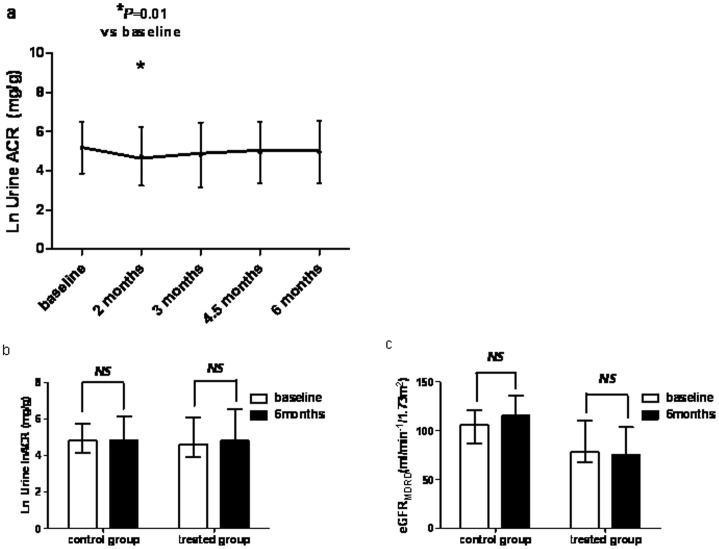
Urine albumin-to-creatinine ratio (ACR) in the treatment. a: The ACRs in the treated group (n = 22) at 2-, 3-, 4.5-, and 6-month follow-up assessments. Error bars represent 95% confidence intervals. b: The ACRs in the treated and control group before and after follow up. c: The eGFR_MDRD_ in the treated and control group before and after follow up.

The ACR decreased from 97.39 mg/g (62.43–476.70) to 71.65 mg/g (40.40–469.98) at 2 months (P = 0.01) and 120.36 mg/g (33.89–695.26) at 6 months (P = 0.239, Figure 4 a).

Comparison of outcome measures between before and after treatment of longitudinal study is shown in Table 4. After 6-month of cholecalciferol treatment, HDL-c was significantly higher than that at baseline, while there was no difference in control group. On the contrary, PTH was significantly increased after 6-month follow up in control group, while there was no change in treated group. There was no significant change in UACR and eGFR_MDRD_ before and after follow up both in control and treated group. (Figure 4 b and c).

**Table 4:** General and clinical parameters of follow-up in longitudinal study.

Treated group (n = 22)				Control group(n = 24)				
	baseline	6 months	P value		baseline	6 months	P value	
**HbA_1_C(%)**	7.1±1.4	7.2±1.4	0.86		8.2±1.3	8.2±1.5	0.80	
**SBP(mmHg)**	138±21	135±12	0.38		140±21	140±19	0.92	
**DBP(mmHg)**	79±10	76±9	0.23		81±7	78±9	0.06	
**TC(mmol/l)**	5.05±1.1	5.5±1.4	0.06		4.87±1.2	5.4±0.9	0.01	
**TG(mmol/l)**	2.31±1.3	1.79±0.95	0.05		2.17±2.0	2.13±2.6	0.92	
**HDL-c(mmol/l)**	1.08±0.3	1.21±0.24	0.02		1.16±0.32	1.21±0.29	0.25	
**LDL-c(mmol/l)**	2.82±0.7	3.18±0.88	0.06		2.63±0.77	3.07±0.81	0.00	
**Ca(mmol/ml)**	2.32±0.1	2.35±0.1	0.66		2.25±0.1	2.28±0.9	0.17	
**Pi(mmol/ml)**	1.29±0.2	1.29±0.2	0.95		1.26±0.17	1.18±0.11	0.01	
**PTH(ng/l)**	33.5±15.5	32.09±11.04	0.50		30.82±9.40	45.32±24.03	0.004	

### Vitamin D Concentration Increased Significantly in the Cholecalciferol Treated Group

**Figure 5:**
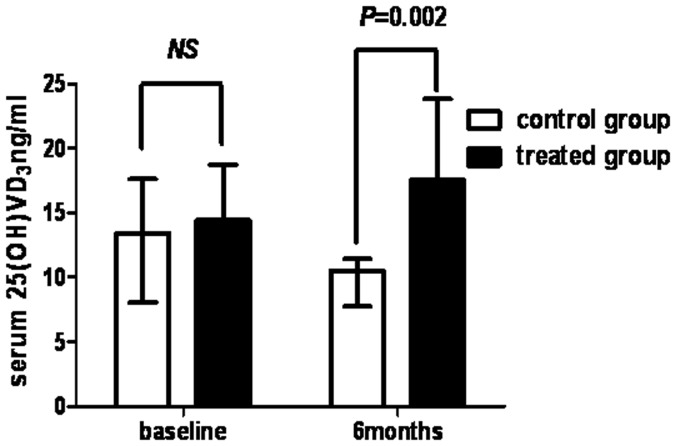
Changes in serum 25(OH)D_3_ levels between the groups. Serum 25(OH)D_3_ levels at baseline and at the 6-month follow-up assessment in the treated group and in the control group. Error bars represent 95% confidence intervals.

There was no significant difference in serum 25 (OH)D_3_ levels at baseline between the treated group and the control group. However, levels in the treated group were significantly higher than those in the untreated group (17.56 (12.23, 23.83) ng/ml vs. 10.52 (7.75, 11.42) ng/ml, P = 0.002) at 6 months (Figure 5).

### Inverse Trend between uACR and Serum 25(OH)D_3_ Levels

**Figure 6:**
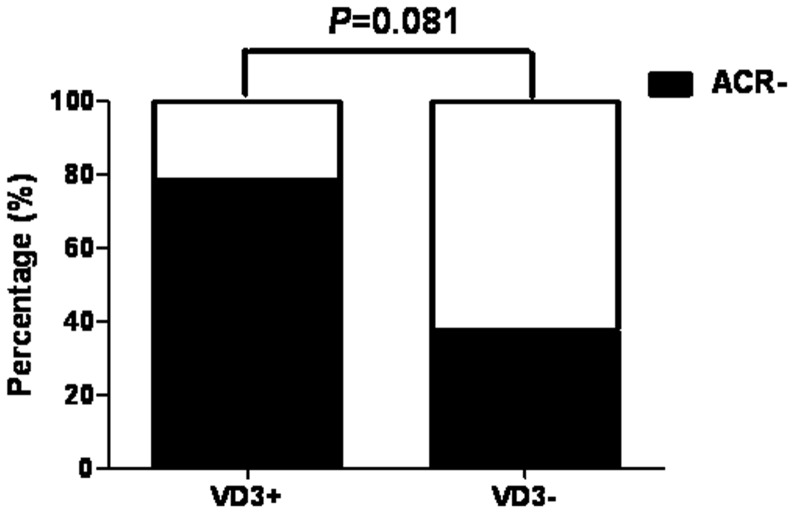
Different treatment effects in vitamin D treated groups. VD3+ and VD3− reflect the rise and fall of serum 25(OH)D_3_ levels after treatment with cholecalciferol.▪ACR- reflect the decrease of urinary ACR after treatment with cholecalciferol.

The results above prompted us to explore the relationship between uACR and serum vitamin D concentrations in the treated group. The patients in treated group with increased serum 25(OH)D3 levels (VD3+, Figure 6) after 6 months’ treatment, had higher percentage of ACR decreasing (ACR-) than those without increased serum 25(OH)D3 levels (VD3−, Figure 6), although the results of Fisher’s Exact test did not reveal a significant difference (P = 0.081).

### Changes in Serum Calcium and Phosphate During Follow-up

Vitamin D is important in electrolyte regulation. There was no significant difference in serum calcium and phosphate during the entire follow-up period in the treated group. At baseline, the serum calcium concentration was 2.32±0.1 (mmol/l) and the serum phosphate concentration was 1.29±0.2 (mmol/l), while at 6 months, the corresponding concentrations were 2.35±0.1(mmol/l) and 1.29±0.2 (mmol/l), respectively (P = 0.66, 0.95, respectively).

## Discussion

This is the first study to demonstrate that serum vitamin D concentrations are significantly lower in diabetic patients with macroalbuminuria, but not in diabetic patients with microalbuminuria. Furthermore, nutritional vitamin D, cholecalciferol at a conventional dose, may play an important role in terms of an antiproteinuric effect in Chinese patients with T2DM. In addition, observed improvements in proteinuria were associated with a rise in vitamin D concentrations.

Vitamin D is known for its role in the regulation of calcium and phosphate, but recent research has revealed its indispensable role also in the regulation of renal function. Preclinical studies have demonstrated the renoprotective function of vitamin D [9]–[12], and clinical trials are currently investigating this further. In our study, we found that serum vitamin D levels were significantly decreased, and were independently correlated with uACR in patients with DN.

Vitamin D analogs, mainly paricalcitol, can effectively decrease proteinuria [6]. However, this agent is too expensive to be used widely. In our study, we found that a conventional dose of cholecalciferol 800 units daily had a similar effect to that of vitamin D analogs. The ACR in the treated group had decreased significantly by the 2-month follow-up assessment. This result is consistent with those of the VITAL study, which demonstrated an additional reduction in albuminuria when an active vitamin D analog was used in conjunction with RAAS inhibition [6]. Serum 25(OH)D_3_ levels in the treated group increased significantly compared to the control group at 6 months. In addition, patients in the treated group who experienced an increase in serum 25(OH)D_3_ levels had a greater improvement in the ACR, while those with decreased 25(OH)D_3_ levels were more likely to have poor ACR results at 6 months.

In addition, Kim et al. reported that high doses of nutritional vitamin D (40000 units weekly), cholecalciferol, can reduce proteinuria in patients with diabetes [7] either. However, high doses of vitamin D are commonly associated with adverse events especially in long-term treatment. Most of the patients with diabetic nephropathy require long-term medication, while the long term safety of such large dose of vitamin D treatment hasn’t been tested before. In our study, the long-term safety of 800 units daily of cholecalciferol which is the recommended by Chinese Nutrition Society for Chinese, has been confirmed. Even more the effect of low dose of cholecalciferol was similar with that of large dose of cholecalciferol.

The anti-proteinuric effect of vitamin D in DN is due to its ‘non-classical’ effects, which are unrelated to its role in mineral metabolism, the classical vitamin D effect. The ‘non-classical’ effects are mediated by VDR activation [11]. Evidence suggests that the effect of VDR activation is partly that of negatively regulating RAAS, which plays a critical role in the development of DN [11]–[13]. Zhang et al. showed that, in VDR knockout mice, increased levels of renin, angiotensinogen, transforming growth factor-β (TGF-β), and connective tissue growth factor, accompany severe renal injury. The same group also showed that combination therapy with an AT1 blocker and vitamin D analogs markedly ameliorated DN in experimental animal models, and the effects of combined therapy were better than those observed with either agent alone. Vitamin D blocks the compensatory renin increase caused by RAAS inhibitors. The VITAL study demonstrated similar effects to those observed in animal studies, with combined therapy with paricalcitol and RAAS inhibition in patients with DN [12].

Unexpectedly, the anti-proteinuric effect had disappeared at the 6-month follow-up assessment in the treated group, although there was a trend for lower ACR levels than at baseline in the treated group, or even at the end of the 6-month period, and also for lower ACR levels in the treated group than in the control group. However, these differences were not significant. In fact, in the VITAL trial, although the reduction in the ACR was sustained during the entire treatment phase both in the 1 µg and 2 µg paricalcitol groups, a peak occurred at the third month, and thereafter the ACR in both groups revealed a marked improvement [6]. This indicates that the actual dose was not implicated. In the cholecalciferol trial, a significant reduction in the ACR with treatment occurred at 2 months, [7] at 2 months, while albuminuria also restored at 4 months. Thus, it appears that the antiproteinuric effect is greatest during the first months of treatment, but may not be significant after this.

The likeliest explanation for this phenomenon may lie with 24-hydroxlayse. On the one hand, vitamin D analogs or nutritional cholecalciferol up-regulate serum vitamin D levels, but on the other hand, 24-hydroxlayse is dramatically activated, thus causing an increasing deactivation of active vitamin D [13]. As Helvig suggests, dysregulation of CYP24 may be a major factor contributing to both vitamin D insufficiency and resistance to vitamin D therapy in CKD [14]. A further explanation may lie with vitamin D binding protein (VDBP), which has a role both in maintaining total levels of vitamin D and in regulating the amounts of free (unbound) vitamin D that are available for specific tissues and cell types. Various physiological conditions affect VDBP levels, with the nephritic syndrome, for example, causing a loss of VDBP [15]. It appears that levels of plasma VDBP can be upregulated following cholecalciferol treatment [7].

Diabetic nephropathy is characterized by persistent albuminuria. However, not all the patients with microalbuminuria will progress to diabetic nephropathy [16].In our study, serum 25(OH)D_3_ concentrations were significantly correlated with ACRs in patients with DN, while serum 25(OH)D_3_ levels were not significantly lower in the MA group than those in either the control or the NA groups. To date, no other study has reported similar results. Low levels of,serum 25(OH)D_3_ might be a indicator of DN in the early stage. Further studies are needed to be done on this point.

We also found that mean 25(OH)D_3_ concentrations were significantly lower in females than in males, in our cross-sectional study, which was consistent with previous studies that demonstrated a stronger association between low vitamin D levels and females [5], [17]. Gender differences also appeared in the multiple linear stepwise regression equation, and a low vitamin D status was more closely associated with micro-albuminuria in males than in females.

Limitations of this study need to be considered. Both in the cross-sectional study and in the longitudinal study, the sample size was small. In addition, the follow-up period was short. As enrollment and the follow-up periods mostly occurred in wintertime, this might affect vitamin D concentrations, and might also influence treatment outcome. Discrepancy in the responsiveness to UVB radiation is clear between individuals [18], a factor that is very difficult to control. In addition, we did not include a dose gradient, and the best effective dose needs further exploration. Finally, we were unable to define a difference in efficacy between micro- and macro-albuminuria, due to the small population. The limitations mentioned above are important when considering future clinical trials with cholecalciferol.

### Conclusions

Low vitamin D levels are common in patients with T2DM and albuminuria, particularly those with macro-albuminuria. Conventional doses of cholecalciferol, 800 IU, may have antiproteinuric effects on Chinese patients with DN.
